# An analysis of 3D knee kinematic data complexity in knee osteoarthritis and asymptomatic controls

**DOI:** 10.1371/journal.pone.0202348

**Published:** 2018-10-01

**Authors:** Neila Mezghani, Imene Mechmeche, Amar Mitiche, Youssef Ouakrim, Jacques A. de Guise

**Affiliations:** 1 LICEF Research center, TÉLUQ University, 5800 Rue Saint-Denis, Montreal (QC), H2S 3L4, Canada; 2 INRS - Centre énergie, matériaux et télécommunications, 800 Rue de la Gauchetiére West, Montreal (QC), H5A 1E4, Canada; 3 Laboratoire de recherche en imagerie et orthopédie, Centre de recherche du CHUM, École de technologie supérieure, 900 Rue Saint-Denis, Montreal (QC), H2X 0A9, Canada; University of Umeå, SWEDEN

## Abstract

Three-dimensional (3D) knee kinematic data, measuring flexion/extension, abduction/adduction, and internal/external rotation angle variations during locomotion, provide essential information to diagnose, classify, and treat musculoskeletal knee pathologies. However, and so across genders, the curse of dimensionality, intra-class high variability, and inter-class proximity make this data usually difficult to interpret, particularly in tasks such as knee pathology classification. The purpose of this study is to use data complexity analysis to get some insight into this difficulty. Using 3D knee kinematic measurements recorded from osteoarthritis and asymptomatic subjects, we evaluated both *single feature complexity*, where each feature is taken individually, and *global feature complexity, where features are considered simultaneously*. These evaluations afford a characterization of data complexity independent of the used classifier and, therefore, provide information as to the level of classification performance one can expect. Comparative results, using reference databases, reveal that knee kinematic data are highly complex, and thus foretell the difficulty of knee pathology classification.

## Introduction

Three-dimensional (3D) knee kinematic data, measuring knee flexion/extension, abduction/adduction, and internal/external rotation, are increasingly used in gait analysis towards quantifying the knee function [1], understanding pathological knee alterations [2], and assessing the progression of knee pathologies and their impact on the gait [3, 5]. They also offer opportunities for diagnostics [6], classification [7], and therapy of knee musculoskeletal pathologies [8]. Kinematic data are generally given in the form of normalized curves of measurements recorded on a treadmill at regular intervals of time during a gait cycle. Several studies have used knee kinematic data to obtain crucial information about musculoskeletal pathologies [9], to distinguish between knee osteoarthritis and asymptomatic subjects [10], and to further classify osteoarthritis populations with respect to the pathology level of severity [4, 7, 11, 12]. These studies have generally reported, without mentioning specifically the gender corresponding to data measurements, that the high dimensionality of kinematic data, their significant intra-class variability and interclass proximity, usually make their analysis a difficult task. However, no study has attempted to explain this difficulty in terms of objective metrics of data complexity. Instead, informal assessments have been generally invoked to deem knee kinematic data as “simple” or “complex,” using classification rates obtained with a common classifier believed to be appropriate for the type of application at hand [7, 11]. Although such a characterization can be sometimes useful, it does not afford a general, objective, and practical means of assessing the complexity of the data. In this study, we use classifier independent data complexity metrics to evaluate how difficult it can be to classify knee kinematic data.

The design and study of data complexity metrics is an emergent subject in data mining and classification. In this context, complexity measures that correlate linearly with classification error have been investigated in [13, 14]. By examining the performance of several classifiers in a space defined by complexity measures, the studies in [15, 16] were able to build a model of a classifier accuracy based on the data complexity measures divided into three general types: (1) class ambiguity, to characterize the overlap of classes in feature space. This is in general due to class representation features that cause ambiguous interpretations. The study [17] gives a lower bound on the achievable error rate in this case; (2) boundary complexity, related to the Kolmogorov complexity [18, 19], defined as the minimum length of a computer program needed to reproduce the class boundary: a classification problem is complex if it takes a “long” algorithm to describe the class boundary. Because the Kolmogorov complexity is algorithmically uncomputable [20], approximate measures are used which describe geometrical properties of data sets. Such measures, which depend on the data set only, can guide the selection of appropriate classifiers; and (3) geometry and density of manifolds, which describe sample sparsity, and feature space dimensionality.

This study investigates such complexity metrics to evaluate knee kinematic data complexity, and does so for both *single features*, i.e., features considered individually, and *global features*, i.e., for multiple features taken simultaneously. The data describe the temporal variation of the three dimensions of knee rotation during locomotion, namely knee flexion/extension, abduction/adduction, and internal/external, in pathology classification problems involving knee osteoarthritis. Osteoarthritis (OA) is the most common type of musculoskeletal disorder and the knee is one of the most affected joints [21]. The kinematic data have interesting characteristics: (i) they reflect the functional status of the knees, (ii), they are objective non-invasive measurements and non-radiant like conventional static X-ray imaging and, (iii) they can be used further by health care professionals to personalize, and therefore enhance, care pathways of knee OA patients. Traditionally, kinetic (rather than kinematic) variables, the external knee adduction moment in particular, have been used in the assessment of the progression and severity of knee OA. However, their measurement needs sophisticated setups, which are, generally, available only in specialized gait study laboratories (Simon 2004). Kinematic data, instead, are acquired in a normal clinical setting, using a commercially available treadmill and a simple non invasive knee attachment system.

## Materials and methods

### Knee kinematic data collection

**Fig 1:**
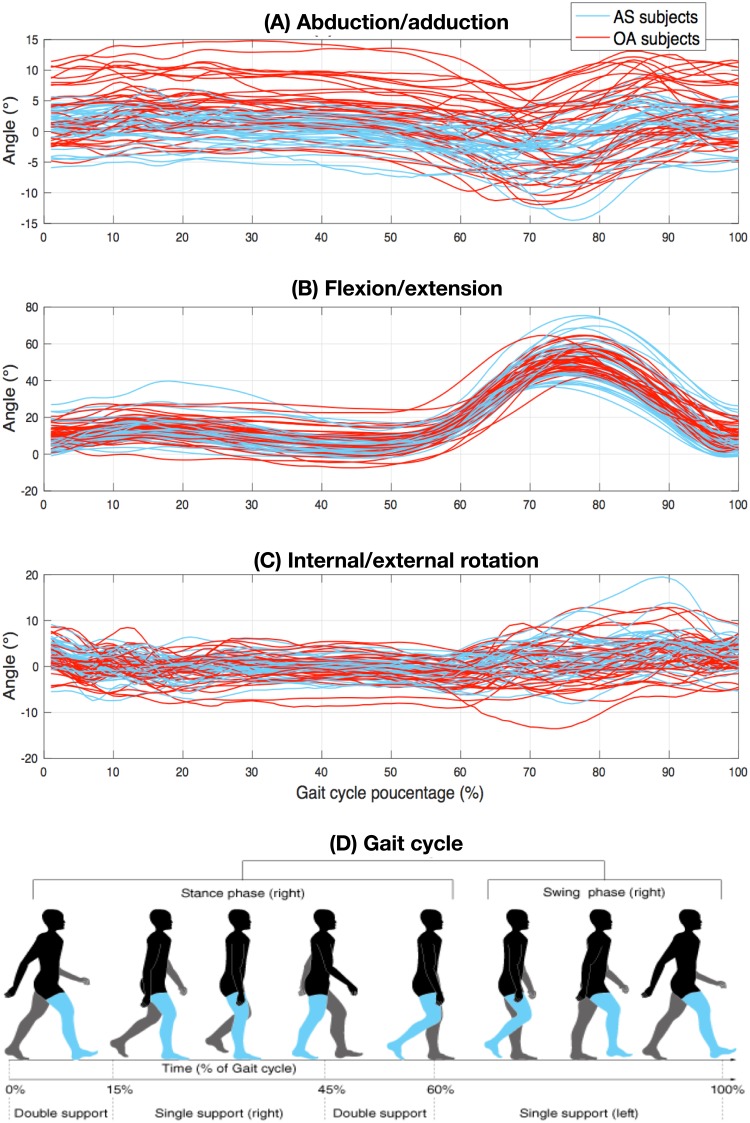
Measures of class overlap in feature space: (A) Abduction/adduction (B) Flexion/extension, and (C) Internal/external rotation. The signals were interpolated and resampled from 1% to 100% (100 points) of the gait cycle. Each red curve represents an OA subject and each blue curve represents an AS subject (D) The gait cycle phases.

Analysis of 3D knee kinematic data measurements, consisting of the temporal variation of the three dimensions of knee rotation, namely the rotation angles measured in the sagittal, frontal, and transverse planes during a full gait cycle (Fig 1), serve knee pathology classification algorithms [22] and can subsequently guide OA therapy plans [8].

The data used in this study was collected, for research projects [3, 23, 24], using a state-of-the-art KneeKG acquisition system. The accuracy and reproducibility of the system have been assessed in several studies [25–27]. For each subject, the positional angles are recorded during two walking trials of 25 sec on a treadmill. For each participant patient, current methods of analysis have used the average of about a dozen curves in the set of measurement curves, so chosen as to maximize a correlation criterion between classes, namely the Inter Class Correlation Index of the observations [28]. In our study, a series of fifteen (15) full gait cycle walks on the treadmill are averaged to obtain a representative mean pattern per subject based on an adjusted coefficient of multiple determinations [29]. This is followed by interpolation and re-sampling from 1% to 100% of the gait cycle, therefore giving 100 measurement points for each participant. This yields a significant amount of high-dimensional data, leading to “the curse of dimensionality,” in classification [30]. Also, knee pathologies notoriously cause high within-class variability and in-between class similarity (example in Fig 1).

The data collection was approved by institutional ethics committees of the University of Montreal Hospital Research Center (Reference numbers CE 10.001-BSP and BD 07.001-BSP) and of the École de technologie supérieure (Reference numbers H20100301 and H20170901). All subjects provided written informed consent before the studies began. We used 3D knee kinematic data from two groups. The first group included 40 patients with knee OA diagnosed according to clinical and radiographic criteria defined by Altman et al. [31]. The patients were excluded if they had vestibular, neurological, or musculoskeletal disorders, fracture of the lower extremity, rheumatoid arthritis, or generalized osteoarthritis, limping gait or any condition that could affect a treadmill walking evaluation. The second group, which formed the control group, contains 40 asymptomatic participants (AS). Table 1 summarizes the participants’ demographic characteristics and their walking speed. A statistical *t*-test was performed to compare the two groups characteristics using SPSS 18.0 (Statistical Package for Social Sciences) (SPSS Inc. Released, 2009. PASW Statistics for Windows). A threshold of significance set at *p* = 0.05 shows that there is no statistical difference between the two groups except for the height variable.

**Table 1:** General subject characteristics.

	OA group	AS group
	N = 40	N = 40
Age (year)	62.4 ± 8.2	48.6 ± 17.47
Height(m)	1.61* ± 0.09	1.67 ± 0.10
Weight (kg)	82.28 ± 18.84	69.70 ± 11.87
BMI (kg / m2)	31.20 ± 5.59	24.69 ± 3.05
Walking speed (m/s)	1.31 ± 0.96	2.47 ± 1.42
Proportion of women	72%(24)	16%(10)

* Significant difference between groups with a p < 0.05

### Features Extraction on 3D Knee kinematic data

A set of 62 biomechanical features are extracted from these 3D kinematic patterns for data complexity analysis. The features chosen are biomechanical parameters routinely assessed in clinical biomechanical studies of knee OA populations; for instance, maximum, minimum, varus and valgus thrust, angles at initial contact, mean values and range of motion (ROM) throughout gait cycles or specific gait sub-cycles (i.e., loading, stance, swing, etc.) [2, 3, 32]. The 3D knee kinematic data were normalized to remove dependence on magnitude and scale.

### Knee kinematic data complexity

Data complexity is evaluated on the descriptive biomechanical features according to both single features and global features complexity metrics. The first metric evaluates the overlap of individual feature measurements whereas the latter evaluates the separability of classes and the geometry of the manifolds spanned by each class when the features are considered simultaneously.

#### Single feature complexity

Complexity of a single feature informs us on that particular feature discriminant power, i.e., its capacity to distinguish data samples from distinct classes. Such a characterization is useful for clinical assessments. Indeed, it allows the study of specific kinematic points of interest, having a clinical meaning, in order to correlate them with typical knee function behavior.

For each individual feature, the following three metrics examine the range and spread of the values of instances from distinct classes. Specifically, let $D=\{x_{1},x_{2},.....,x_{n}\}$ be a data set containing *n* knee kinematic data measurements, each belonging to one of two distinct classes *c*_1_ and *c*_2_. Each element **x**_*i*_ is characterized by a feature vector (*f*_1_, *f*_2_, …*f*_*p*_), where *p* is the dimension of the feature space. In our case *p* = 62. The complexity metrics are:

- Fisher discriminant ratio (F1): for a two-class data set, ratios F1 = {F1_1_, F1_2_, …, F1_*p*_} for feature *f*_*i*_, (*i* = 1, …, *p*), are defined as:
  $$\begin{array}{c} F1_{i}=\frac{{\left(\mu_{i1}-\mu_{i2}\right)}^{2}}{\left(\sigma_{i1}^{2}+\sigma_{i2}^{2}\right)}, \end{array}$$ (1)
  where *μ*_*i*1_, *μ*_*i*2_, *σ*_*i*1_, *σ*_*i*2_ are the means and variances of the two classes, respectively, according to the *i*^*th*^ feature, (*i* = 1, 2, …, *p*). The range of F1_*i*_ is [0, +∞[. Small values indicate strong class overlap. High values indicate good class separation when using the *i*^*th*^ feature.
- Volume of overlap region (F2): This measure estimates the amount of relative overlap of the bounding regions of two classes [33]. Let max_*i*1_ = max(*f*_*i*_, *c*_1_), max_*i*2_ = max(*f*_*i*_, *c*_2_), min_*i*1_ = min(*f*_*i*_, *c*_1_), min_*i*2_ = min(*f*_*i*_, *c*_2_) the maximum and minimum values of each feature *f*_*i*_ in the classes *c*_1_ and *c*_2_. For two-class data sets, the ratio F2_*i*_ for each feature *f*_*i*_, (*i* = 1, 2, …, *p*) is computed as:
  $$\begin{array}{c} \text{F}2_{i}=\frac{\text{min}\left({\text{max}}_{i1},{\text{max}}_{i2}\right)-\text{max}\left({\text{min}}_{i1},{\text{min}}_{i2}\right)}{\text{max}\left({\text{max}}_{i1},{\text{max}}_{i2}\right)-\text{min}\left({\text{min}}_{i1},{\text{min}}_{i2}\right)} \end{array}$$ (2)
  The range of F2 is [0, 1]. Larger values indicate larger class overlap, and higher data complexity thereof. A shortcoming of this measure is that a single feature with non-overlapping regions causes F2 to be zero.
- Feature efficiency (F3): this measure is particularly relevant when dealing with high-dimensional data. It informs on how much each feature contributes to the separation of the classes, and the contribution is called efficiency. For each feature, the ambiguous (overlapping) regions are removed so that only non-overlapping regions remain for consideration.
  Let MinMax_*i*_ = min(max(*f*_*i*_, *c*_1_), max(*f*_*i*_, *c*_2_)) and MaxMin_*i*_ = max(min(*f*_*i*_, *c*_1_), min(*f*_*i*_, *c*_2_)). For each feature *f*_*i*_, let *Q*_*i*_ be the following ratio:
  $$\begin{array}{c} \text{F}3_{i}=\frac{|f_{i}\in \left[{\text{MinMax}}_{i},{\text{MaxMin}}_{i}\right]|}{n}, \end{array}$$ (3)
  where | | denotes the number of non-overlapping elements and *n* is the total number of elements in both classes. When there is no overlap between classes, F3_*i*_ achieves the maximum value of 1.

Each single feature complexity metric requires the estimation of high and low thresholds to assess whether the feature is complex or not. We estimated the various thresholds based on the probability distributions of the feature complexity measures. For each metric, i.e., the Fisher discriminant ratio F1_*i*_, the volume of overlap region F2_*i*_, and the feature efficiency F3_*i*_, the best-fitting distribution is determined by the maximum likelihood estimation. The thresholds are subsequently estimated using the rule of thumb of 5th and 95th quantiles of the probability distribution.

#### Global feature complexity

The global feature complexity is based on the following metrics: fraction of points on the class boundary (N1), ratio of average intra/inter class nearest neighbor distance (N2), error rate of a nearest neighbor distance (N3), fraction of maximum covering spheres (T1), average number of points per dimension (T2).

- Fraction of points on the class boundary (N1): This measure estimates the length of the class boundary. The minimum spanning tree (MST) connecting all the training samples is generated and the fraction of points of a class connected to the opposite class is computed [34, 35]. The range of *N*1 is [0, 1]. Large values indicate that the majority of points lay closely to the class boundary, which can make it difficult to locate the class boundary accurately.
- The ratio of average intra/inter class nearest neighbor distance (N2): Let **x**_*i*_ denote an input sample, *i* = 1, 2, …, *n*, where *n* is the number of samples. Let IntraDist(**x**_*i*_) designate the distance between **x**_*i*_ and its nearest neighbor within the class, and InterDist(**x**_*i*_) the distance to its nearest neighbor of the other class. The ratio of average intra/inter class nearest neighbor distance (N2) is the ratio of the sum of the intra-class distances to the sum of the inter-class distances for each input example:
  $$\begin{array}{c} N2=\frac{\sum_{i=0}^{n}\text{IntraDist}\left(x_{i}\right)}{\sum_{i=0}^{n}\text{InterDist}\left(x_{i}\right)} \end{array}$$ (4)
- Leave-one-out error rate of the Nearest neighbor classifier (N3): This measure informs on how close the samples of different classes are. It returns the leave-one-out error rate of the Nearest neighbor classifier.
- Nonlineality of the Nearest neighbor classifier (N4): This measure creates a test set and computes the test error of the Nearest neighbor classifier. The range of N4 is [0, 1]. Low values indicate low error by linear separation.
- Fraction of maximum covering spheres (T1): This measure describes the shapes of class manifolds using the notion of adherence subset [36]. An adherence subset is a sphere centered on an sample of the data set which is grown as much as possible before touching any sample of another class. The T1 is measured based on the number of spheres normalized by the total number of points.
- Average number of points per dimension (T2): The average number of points per dimension is the ratio of the number of elements in the data set to the number of features. Its value is a rough indicator of sparseness of the data set.

#### Statistical analysis

Following individual feature complexity evaluation, we performed a t-test statistical analysis to assess feature dissimilarity between the two groups (osteoarthritis patients and control participants). Since each feature single complexity variable informs on that particular feature discriminant power, i.e., its capacity to distinguish data samples from distinct classes, our hypothesis is that the features retained by complexity analysis as discriminant are statistically different in the two groups. A P-value of 0.05 was set as the statistical level of significance.

## Results and discussion

We recall that data complexity assessment used 40 osteoarthritis patients (OA) and 40 control participants (AS). A set of 62 biomechanical features (*p* = 62) routinely assessed in clinical biomechanical studies of knee OA populations have been selected (as described in section Features Extraction on 3D Knee Kinematic Data). The analysis was done using Matlab R2017a software (Mathworks, Massachusetts, United State).

### Single feature complexity

The single feature complexity measures have been evaluated using Fisher discriminant ratio (F1), the volume of overlap region (F2), and the individual feature efficiency (F3). The threshold values have been identified, for each complexity metric, using a probability density which best fit the complexity data.

**Fig 2:**
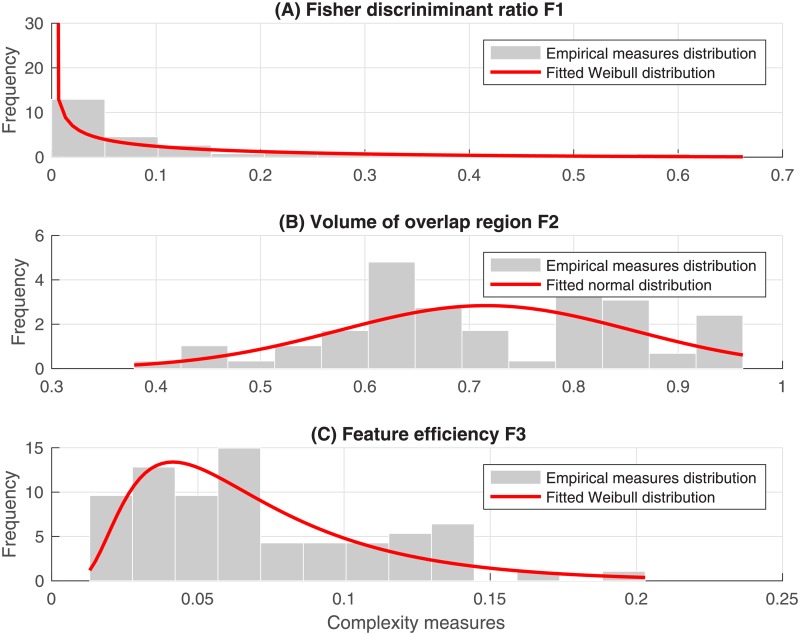
Empirical distribution of the class ambiguity measures (A) The Fisher discriminant Ratio F1, (B) The ratio of the width of the overlap interval F2 and (C) The individual feature efficiency F3. The red curves are the corresponding fitted distribution function.

Fig 2 indicates that the Fisher discriminant ratio F1 and the feature efficiency F2 best follow a Weibull distribution, and the volume of overlap region F3 follows a normal distribution. Table 2 summarizes the thresholds value determined using the 10th and 90th percentiles of each complexity measure empirical density.

**Table 2:** Thresholds for single feature complexity.

Complexity metrics	Low and High thresholds
Fisher’s Discriminant Ratio (F1)	{0.00, **0.33**}
Volume of overlap region (F2)	{**0.47**, 0.99}
Feature efficiency (F3)	{−0.02, **0.13**}

**Fig 3:**
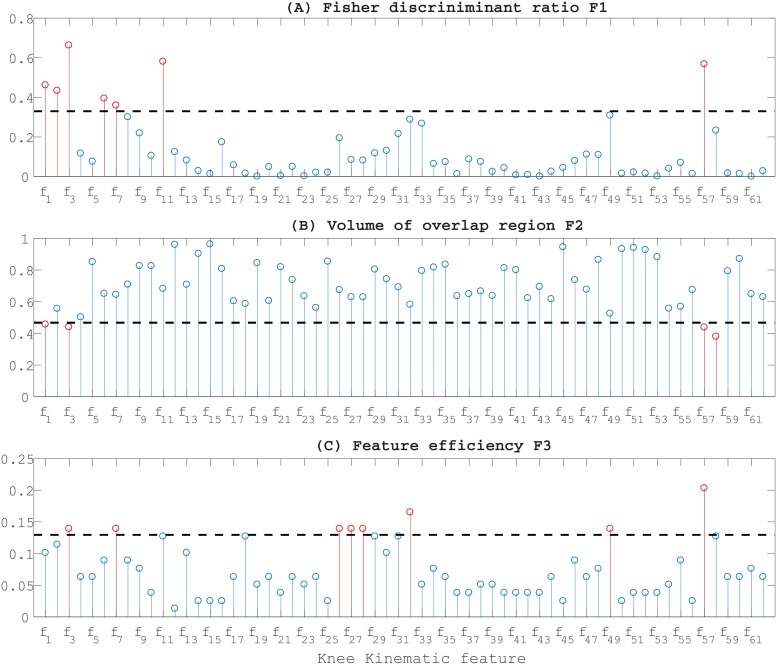
Measures of single feature complexity for feature *f*_*i*_ (*i* = 1, 2, …, 62): (A) The Fisher discriminant Ratio F1_*i*_; a high value of F1_*i*_ indicates that the feature is discriminant. (B) The ratio of the width of the overlap interval F1_*i*_; a low value of F2_*i*_ measure means that the feature can discriminate the samples of different classes. (C) The feature efficiency F3_*i*_; a high value of F3_*i*_ indicates a good efficiency. The horizontal dotted black lines correspond to the threshold values of each single feature complexity. The red vertical lines correspond to the retained discriminant features while the blue ones correspond to the features having high complexity.

The single complexity measures of each feature and the corresponding thresholds are presented in Fig 3. Fig 3A represents the discriminant ratio F1_*i*_ for each feature *f*_*i*_ (*i* = 1, 2, …, 62) computed according to Eq 1. Its threshold was set to 0.33 because we are interested in high values of F1_*i*_ (Table 2, line 1). Except for the set of features {*f*_1_, *f*_2_, *f*_3_, *f*_6_, *f*_7_, *f*_11_, *f*_57_}, the low values of F1_*i*_ demonstrate the low discriminative power of the 3D kinematic features.

In Fig 3B, we represented the ratio of the width of the overlap interval F2_*i*_ of each feature. The threshold here was set to 0.47 because, for this metric, we are interested in low value of F2_*i*_ (Table 2, line 2). The ratio values are generally high except for features {*f*_1_, *f*_3_, *f*_57_, *f*_58_}. Finally, in Fig 3C, we represented the feature efficiency. The low values of F3_*i*_ show that the ratio of the number of samples that are not in the overlapping region to the total number of samples is low except for features {*f*_3_, *f*_7_, *f*_26_, *f*_27_, *f*_28_, *f*_32_, *f*_49_, *f*_57_}.

Although the identified features for each complexity metric are different, there is overlap between some of them. For instance, feature *f*_3_ is discriminant according the three complexity metrics. Same for the feature *f*_1_ et *f*_65_ which is discriminant according both the Fisher discriminant Ratio (F1) and the individual feature efficiency (F3). As a result of the single complexity analysis, the discriminant features are given by the following set of 13 features: {*f*_1_, *f*_2_, *f*_3_, *f*_6_, *f*_7_, *f*_11_, *f*_26_, *f*_27_, *f*_28_, *f*_32_, *f*_49_, *f*_57_, *f*_58_}.

### Statistical analysis

Following the single feature complexity analysis, we performed a statistical *t*−test analysis to examine the differences between the two groups (the osteoarthritis patients (OA) and the control participants (AS)) of these 13 retained features. Table 3 summarizes their clinical meaning and their corresponding *p*-value. The results of the statistical analysis confirms those obtained using the complexity analysis. Indeed, the retained 13 features are statistically significantly different, and can therefore be considered discriminant of OA patients and AS participants (p<0.001).

**Table 3:** Features arising following single feature complexity analysis.

Selected features	*p*-value
*f*_1_: Varus thrust during loading (1% to 20% of GC)	0.000
*f*_2_: Varus thrust during loading (1% to 10% of GC)	0.000
*f*_3_: Varus thrust during loading (1% to 30% of GC)	0.000
*f*_6_: Mean abduction angle during stance phase	0.000
*f*_7_: Mean abduction angle during mid stance and terminal stance phase	0.001
*f*_11_: Valgus thrust during loading (1% to 30% of GC)	0.000
*f*_26_: Maximum of the adduction/abduction angle	0.01
*f*_27_: Minimum of the adduction/abduction angle	0.04
*f*_28_: Minimum of the adduction/abduction angle during swing phase	0.04
*f*_32_: Adduction/abduction angle at 20% of GC	0.003
*f*_49_: Adduction/abduction angle absolute value at 20% of GC	0.000
*f*_57_: Range of motion during loading	0.000
*f*_58_: Range of motion absolute value during loading	0.001

Significant difference between groups with a p < 0.05

Both statistical analysis and single complexity analysis support previous studies on biomechanical data and their association with knee pathologies. Indeed, the features *f*_1_, *f*_2_, *f*_3_ and *f*_11_ which are related to the varus or valgus thrust during loading phase, have been identified as the most useful parameter, serving as a diagnostic biomarker [3, 21]. Also the feature *f*_57_, which corresponds to the range of motion during loading phase, has been identified as a part of burden of disease biomarkers to discriminate between moderates OA grades and severe OA grades [3]. Another study compared a set of biomechanical parameters in patients categorized as moderate to severe OA grades [21, 37]. It was reported that both peak knee adduction moment and knee adduction angular impulse increased with knee radiographic grade.

### Global feature complexity

The global feature complexity metrics have been measured using: the Fraction of points on the class boundary (N1), the Ratio of average intra/inter class nearest neighbor distance (N2), the Error rate of a nearest neighbor distance (N3), the Fraction of maximum covering spheres (T1), the Average number of points per dimension (T2).

Table 4 summarizes these metrics. It shows high values of the ratio of average intra/inter class nearest neighbor distance (N2 = 0.95); this indicates that samples from the same class are dispersed. This result is confirmed by the fraction of points on the class boundary (N1 = 0.54) which shows that an important number of samples are located near the class boundaries. As a result the nearest neighbor error rate (N3 = 0.42) approaches that of random classification. Moreover, the analysis of the global complexity metrics shows the sparsity of the features. Indeed, the fraction of maximum covering spheres is equal to 1 (T1 = 1), which means that the largest adherence subset is one sample. Moreover, the average number of points per dimension (T2 = 1.29) indicates high sparseness of the data set. This sparsity is an important problematic in classification [30].

**Table 4:** Global complexity metrics of 3D knee kinematics.

Complexity measures	Values
Fraction of points on the class boundary (N1)	0.5
Ratio of average intra/inter class nearest neighbor distance (N2)	0.95
Error rate of a nearest neighbor distance (N3)	0.36
Non-linearity of 1-nearest neighbor classifier (N4)	0.20
Fraction of maximum covering spheres (T1)	1
Average number of points per dimension (T2)	1.29

Table 1, which describes the general subject characteristics, shows that the proportion of women with respect to that of men is higher in the OA group (72% of women against 38% of men) while it is lower in the AS group (16% of women against 84% of men). For further analysis, to take into account gender, we measured the data global complexity for men and women separately to determine if it is gender-dependent. Table 5 summarizes the complexity results per gender. Overall, global complexity is high in both disease groups when analyzed separately per gender, thus confirming the conclusions drawn from Table 4. Moreover, the complexity is higher for women than men. This explains recent literature [38, 39] reporting more effective automatic classification in men’s groups than in women’s.

**Table 5:** Global complexity metrics of 3D knee kinematics according the gender.

Complexity measures	Men group	Women group
Fraction of points on the class boundary (N1)	0.42	0.55
Ratio of average intra/inter class nearest neighbor distance (N2)	0.87	0.93
Error rate of a nearest neighbor distance (N3)	0.24	0.42
Non-linearity of 1-nearest neighbor classifier (N4)	0.06	0.20
Fraction of maximum covering spheres (T1)	1	1
Average number of points per dimension (T2)	0.47	0.68

### Comparison to the complexity of other types of data

We compared the knee kinematic data complexity measures to those obtained using the data in two public databases for classification tests: the *Wind database*, which contains 178 samples from two classes described by 13 features, and the *IRIS database*, which contains 150 samples from two classes described by 4 features (Table 6).

**Table 6:** Complexity metrics of 3D knee kinematics and reference databases.

Complexity measures	3D knee kinematics	Wine	Iris
Fraction of points on the class boundary (N1)	0.5	4.7 10^−4^	0.6
Ratio of average intra/inter class nearest neighbor distance (N2)	0.9	0.026	0.1
Error rate of a nearest neighbor distance (N3)	0.3	2.3 10^−4^	0
Non-linearity of 1-nearest neighbor classifier (N4)	0.2	0.0	0
Fraction of maximum covering spheres (T1)	1	1	1
Average number of points per dimension (T2)	1.29	522	37.5

The ratio of average intra/inter class Nearest neighbor distance (N2) shows that the discriminant power is higher for Wine and IRIS than for the knee kinematic data. Indeed, N2 is 0.9 for the kinematic data, 0.026 for Wine, and 0.1 for IRIS. Also, the average number of points per dimension (T2) is very low for knee kinematic data. This indicates that 3D knee kinematic data is significantly more complex compared to that of IRIS and Wind data.

## Conclusion

This study investigated 3D knee kinematic data complexity using both single feature and global feature complexity metrics. The first evaluates the overlap of individual feature measurements, whereas the latter measures the separability of classes and the geometry of the manifolds spanned by each class when the features are considered simultaneously. The results reveal that knee kinematic data are sparse and highly complex and, thus, foretell the difficulty of knee pathology classification. Indeed, from the initial set of 62 biomechanical features, only 13 features have been identified as not complex. Such information might be useful in future work (1) in the selection of an appropriate classification algorithm and for the interpretation of classification rates and, (2) in clinical studies to identify biomarkers for knee pathologies.

## References

[1] Clement J, Toliopoulos P, Hagemeister N, Desmeules F, Fuentes A, Vendittolo PA. Healthy 3D knee kinematics during gait: differences between women and men, and correlation with x-ray alignment. Gait and Posture. 2018;. 10.1016/j.gaitpost.2018.06.024 29933182

[2] Bytyqi D, Shabani B, Lustig S, Cheze L, Karahoda Gjurgjeala N, Neyret P. Gait knee kinematic alterations in medial osteoarthritis: three dimensional assessment. Int Orthop. 2014;38(6):1191–1198. 10.1007/s00264-014-2312-3 24619388 PMC4037512

[3] Mezghani N, Ouakrim Y, Fuentes A, Mitiche A, Hagemeister N, Vendittoli P, et al Mechanical biomarkers of medial compartment knee osteoarthritis diagnosis and severity grading: Discovery phase. J Biomech. 2017;52:106–112. 10.1016/j.jbiomech.2016.12.022 28088304

[4] Mezghani N, Boivin K, Turcot K, Aissaoui R, Hagemeister N, De Guise JA. Hierarchical analysis and classification of asymptomatic and knee osteoarthritis gait patterns using a wavelet representation of kinetic data and the nearest neighbor classifier. Journal of Mechanics in Medicine and Biology, 2008, 8(1), 45–54. 10.1142/S0219519408002474

[5] Zeng X, Ma L, Lin Z, Huang W, Huang Z, Zhang Y, et al Relationship between Kellgren-Lawrence score and 3D kinematic gait analysis of patients with medial knee osteoarthritis using a new gait system. Scientific Reports. 2017;7(1). 10.1038/s41598-017-04390-5 PMC5481437 28642490

[6] Hunter DJ. Focusing osteoarthritis management on modifiable risk factors and future therapeutic prospects. Therapeutic Advances in Musculoskeletal Disease. 2009;1:35–47. 10.1177/1759720X09342132 22870426 PMC3382668

[7] Mezghani N, Ouakrim Y, Fuentes A, Hagemeister N, Aissaoui R, Pelletier M, et al Knee osteoarthritis severity assessment using knee kinematic data classification. Osteoarthritis and Cartilage. 2012;20:S97 10.1016/j.joca.2012.02.102

[8] Gaudreault N, Mezghani N, Turcot K, Hagemeister N, Boivin K, de Guise JA. Effects of physiotherapy treatment on knee osteoarthritis gait data using principal component analysis. Clinical Biomechanics. 2010;. 10.1016/j.clinbiomech.2010.10.004 21071119

[9] Medved V. Measurement of Human Locomotion. CRC Press; 2000.

[10] Beynon MJ, Jones L, Holt CA. Classification of Osteoarthritic and Normal Knee Function Using Three-Dimensional Motion Analysis and the Dempster-Shafer Theory of Evidence. IEEE Transactions on Systems, Man, and Cybernetics, Part A. 2006;36(1):173–186. 10.1109/TSMCA.2006.859098

[11] Mezghani N, Husse S, Boivin K, Turcot K, Aissaoui R, Hagemeister N, et al Automatic classification of asymptomatic and osteoarthritis knee gait patterns using kinematic data features and the nearest neighbor classifier. Biomedical Engineering, IEEE Transactions on. 2008;55(3):1230–1232. 10.1109/TBME.2007.905388 18334419

[12] Fuentes A, Mezghani N, Hagemeister N, de Guise JA. Automatic classification of 3D kinetic data of healthy and ACL dificient participants. In: Orthopaedic Proceedings. vol. 93. Orthopaedic Proceedings; 2011. p. 278–278.

[13] Ho TK. A data complexity analysis of comparative advantages of decision forest constructors. Pattern Analysis & Applications. 2002;5(2):102–112. 10.1007/s100440200009

[14] Bernadó-Mansilla E, Ho TK. Domain of competence of XCS classifier system in complexity measurement space. IEEE Transactions on Evolutionary Computation. 2005;9(1):82–104. 10.1109/TEVC.2004.840153

[15] Ho TK, Bernadó-Mansilla E. Classifier domains of competence in data complexity space In: Data Complexity in Pattern Recognition. Springer; 2006 p. 135–152.

[16] Mansilla EB, Ho TK. On classifier domains of competence. In: Pattern Recognition, 2004. ICPR 2004. Proceedings of the 17th International Conference on. vol. 1. IEEE; 2004. p. 136–139.

[17] Ho TK, Baird HS. Large-scale simulation studies in image pattern recognition. IEEE Transactions on Pattern Analysis and Machine Intelligence. 1997;19(10):1067–1079. 10.1109/34.625107

[18] Kolmogorov AN. Three approaches to the quantitative definition of information*. International Journal of Computer Mathematics. 1968;2(1–4):157–168. 10.1080/00207166808803030

[19] Li M, Vitányi P. An introduction to Kolmogorov complexity and its applications. Springer Science & Business Media; 2009.

[20] Maciejowski J. Model discrimination using an algorithmic information criterion. Automatica. 1979;15(5):579–593. 10.1016/0005-1098(79)90006-2

[21] Sharma L, Song J, Felson DT, Cahue S, Shamiyeh E, Dunlop DD. The role of knee alignment in disease progression and functional decline in knee osteoarthritis. Journal of the American Medical Association. 2001;286(2):188–95. 10.1001/jama.286.2.188 11448282

[22] Hunter D, Williams M, Fuentes A, Makovey J. Utility of mechanical assessment in knee OA management. Osteoarthritis Cartilage. 2013;21:253 10.1016/j.joca.2013.02.524

[23] Boivin K, Hagemeister N, Turcot K, Aissaoui R, Prince F, de Guise JA. Influence of a 3D knee movement analyzer on gait patterns of knee osteoarthritis patients. Gait & Posture. 2006;24:S164–S165. 10.1016/j.gaitpost.2006.11.114

[24] Turcot K, Aissaoui R, Boivin K, Hagemeister N, Pelletier M, de Guise JA. Test-Retest Reliability and Minimal Clinical Change Determination for 3-Dimensional Tibial and Femoral Accelerations During Treadmill Walking in Knee Osteoarthritis Patients. Archives of Physical Medicine and Rehabilitation. 2008;89(4):732–737. 10.1016/j.apmr.2007.09.033 18374005

[25] Lustig S, Magnussen R, Cheze L, Neyret P. The KneeKG system: a review of the literature. Knee Surg Sport Traumatol Arthrosc. 2012;20(4):633–638. 10.1007/s00167-011-1867-4 22215077

[26] Hagemeister N, Parent G, Van de Putte M, St-Onge N, Duval N, de Guise JA. A reproducible method for studying three-dimensional knee kinematics. J Biomech. 2005;38(9):1926–1931. 10.1016/j.jbiomech.2005.05.013 15996675

[27] Sati M, de Guise J, Drouin G. Computer assisted knee surgery: diagnostics and planning of knee surgery. Comput Aided Surg. 1997;2(2):108–123. 9292263 10.1002/(SICI)1097-0150(1997)2:2<108::AID-IGS4>3.0.CO;2-3

[28] Mechmeche I, Mitiche A, Ouakrim Y, De Guise JA, Mezghani N. Data correction to determine a representative pattern of a set of 3D knee kinematic measurements. In: Engineering in Medicine and Biology Society (EMBC), 2016 IEEE 38th Annual International Conference of the. IEEE; 2016. p. 884–887. 10.1109/EMBC.2016.7590842 28268465

[29] Kadaba M, Ramakrishnan H, Wootten M, J JG, Gorton G, Cochran G. Repeatability of kinematic, kinetic, and electromyographic data in normal adult gait. Journal of orthopedic reasearch. 1989;6:849–860. 10.1002/jor.1100070611 2795325

[30] Duda RO, Hart PE, Stork DG. Pattern Classification (2nd Ed). Wiley; 2001.

[31] Altman R, Asch E, Bloch D, Bole G, Borenstein D, Brandt K, et al Development of criteria for the classification and reporting of osteoarthritis. Classification of osteoarthritis of the knee. Diagnostic and Therapeutic Criteria Committee of the American Rheumatism Association. Arthritis Rheum. 2006;29(8):2512–2520. 10.1002/art.1780290816 3741515

[32] Astephen Wilson JL, Deluzio K, Dunbar M, Caldwell G, Hubley-Kozey C. The association between knee joint biomechanics and neuromuscular control and moderate knee osteoarthritis radiographic and pain severity. Osteoarthritis Cartilage. 2011;26(3):186–193. 10.1016/j.joca.2010.10.020 21074628

[33] Lorena AC, de Souto MCP. In: Arik S, Huang T, Lai WK, Liu Q, editors. On Measuring the Complexity of Classification Problems. Cham: Springer International Publishing; 2015 p. 158–167. Available from: 10.1007/978-3-319-26532-2_18.

[34] Friedman JH, Rafsky LC. Multivariate generalizations of the Wald-Wolfowitz and Smirnov two-sample tests. The Annals of Statistics. 1979; p. 697–717. 10.1214/aos/1176344722

[35] Smith SP, Jain AK. A test to determine the multivariate normality of a data set. IEEE Transactions on Pattern Analysis and Machine Intelligence. 1988;10(5):757–761. 10.1109/34.6789

[36] Lebourgeois F, Emptoz H. Pretopological approach for supervised learning. In: 13th International Conference on Pattern Recognition, ICPR 1996, Vienna, Austria, 25-19 August, 1996; 1996. p. 256–260. Available from: 10.1109/ICPR.1996.547426.

[37] Thorp L, Sumner D, Block J, Moisio K, Shott S, Wimmer M. Knee joint loading differs in individuals with mild compared with moderate medial knee osteoarthritis. J Biomech. 2006;54(12):3842–3849. 10.1002/art.22247 17133592

[38] Bensalma F, Dunbar M, Whynot S, Fuentes A, Macdonald H, Ouakrim Y, Richardson G, and Mezghani N. Correlations between kinematics and clinical measures in end-staged knee osteoarthritis patients. P074. In: The 20th Biennial Meeting of the Canadian Society for Biomechanics CSB, 2018, Halifax, Nova Scotia.

[39] Ouakrim Y, Dunbar M, Fuentes A, Whynot S, Macdonald H, Richardson G, and Mezghani N. Classification tool identifying knee arthroplasty candidates based on kinematic data and gender. P064. In: The 20th Biennial Meeting of the Canadian Society for Biomechanics CSB, 2018, Halifax, Nova Scotia.

